# *Rickettsia parkeri* in Argentina

**DOI:** 10.3201/eid1412.080860

**Published:** 2008-12

**Authors:** Santiago Nava, Yasmin Elshenawy, Marina E. Eremeeva, John W. Sumner, Mariano Mastropaolo, Christopher D. Paddock

**Affiliations:** Instituto Nacional de Tecnología Agropecuaria, Santa Fe, Argentina (S. Nava, M. Mastropaolo); Centers for Disease Control and Prevention, Atlanta, Georgia, USA (Y. Elshenawy, M.E. Eremeeva, J.W. Sumner, C.D. Paddock)

**Keywords:** *Rickettsia parkeri*, *Amblyomma triste*, rickettsiosis, Argentina, dispatch

## Abstract

Clinical reports of an eschar-associated rickettsiosis in the Paraná River Delta of Argentina prompted an evaluation of *Amblyomma triste* ticks in this region. When evaluated by PCR, 17 (7.6%) of 223 questing adult *A. triste* ticks, collected from 2 sites in the lower Paraná River Delta, contained DNA of *Rickettsia parkeri*.

Argentina is a large, ecologically diverse country, with at least 10 Neotropical *Amblyomma* tick species that bite humans, including *Amblyomma triste* (*1**,**2*; Figure 1, panels **A**, **B**). Spotted fever group rickettsiae have been identified in 3 *Amblyomma* species in Argentina: *Rickettsia amblyommii* and *R. bellii* in *A. neumanni* ticks from Córdoba Province (*3*), a novel *Rickettsia* sp. in *A. parvum* ticks from Córdoba Province (*4*), and *R. rickettsii* and *R. bellii* in *A. cajennense* ticks from Jujuy Province (*5*). Human diseases in Argentina attributable to tick-borne rickettsiae have been recognized only recently, including several fatal cases of Rocky Mountain spotted fever caused by *R. rickettsii* in Jujuy Province (*5**,**6*), and a milder, eschar-associated, spotted fever rickettsiosis in the Paraná River Delta of Buenos Aires Province (*7*) that closely resembles a newly recognized rickettsial spotted fever in the United States caused by *R. parkeri* (*8*). *R. parkeri* has been detected recently in *A. triste* ticks collected in Uruguay and Brazil (*9**,**10*). We report the occurrence of *R. parkeri* in *A. triste* ticks collected along the Paraná River close to the locations of several recently identified cases of eschar-associated spotted fever.

**Figure 1:**
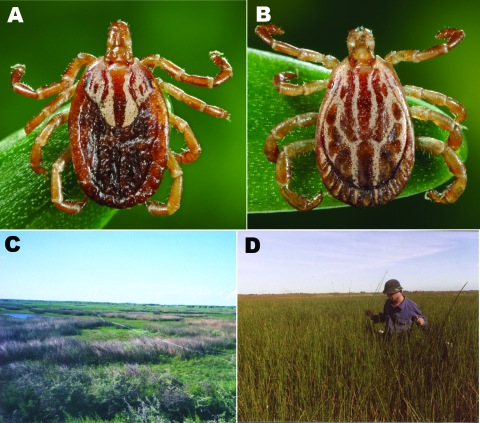
Adult female (A) and male (B) *Amblyomma triste* ticks and tick collection sites in the lower Paraná River Delta of Buenos Aires Province, Argentina, showing freshwater marsh habitats in the Reserva Natural Otamendi (C) and Estación Experimental Instituto, Nacional de Tecnología Agropecuaria, Delta del Paraná (D).

## The Study

**Figure 2:**
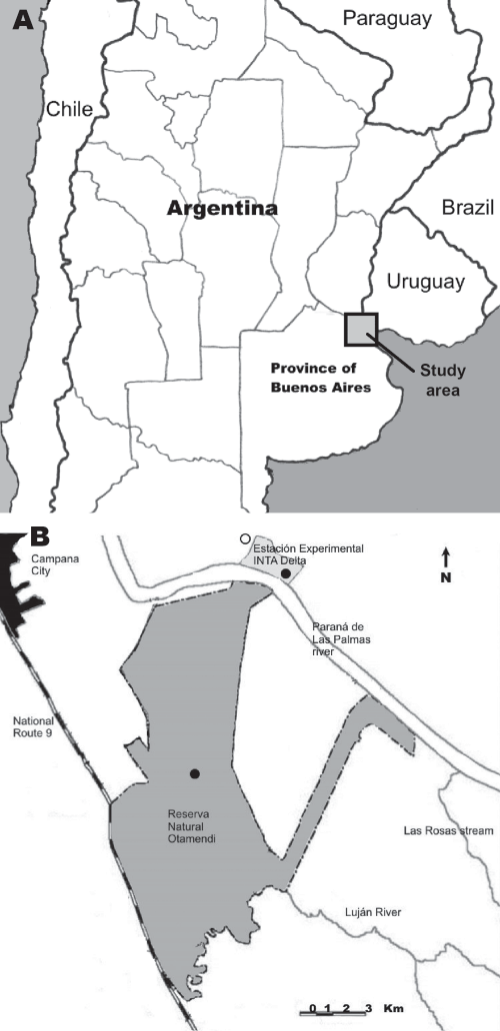
A) Location of study area in the lower Paraná River Delta of Argentina. B) Tick collection sites along the Paraná River (dark circles) and a recently reported case of eschar-associated rickettsiosis (open circle) identified by clinicians in Buenos Aires Province, Argentina (*7*).

Tick collections occurred at 2 sites in Buenos Aires Province, Argentina, during January through December 2007: Reserva Natural Otamendi (34°15′S, 58°52′W) (Figure 1, panel **C**) and Estación Experimental, Instituto Nacional de Tecnología Agropecuaria (INTA), Delta del Paraná (34°11′S, 58°50′W) (Figure 1, panel **D**). Both are located in the lower Paraná River Delta region (Figure 2), which is the southern extension of the Paranense Province of the Amazon Phytogeographic Dominion. The region is characterized by a system of levees that surround temporarily or permanently flooded freshwater marshes (*11*). Humboldt’s willow (*Salix humboldtiana*), Cockspur coral tree (*Erythrina crista-galli*), and *Sapium hematospermum* grow on the levees, and several species of bulrush (*Scirpus giganteus, Schoenoplectus californicus*, *Scirpus americanus*, and *Typha* sp.) and espadaña (*Zizaniopsis bonariensis*) comprise the dominant vegetation in the marshes. Medium to large mammals found at the study sites include wild marsh deer (*Blastocerus dichotomus*), capybara (*Hydrochoerus hydrochaeris*), pampas fox (*Lycalopex gymnocercus*), Geoffroy’s cat (*Oncifelis geoffroyi*), cattle, horses, and dogs.

Questing adult ticks were collected from vegetation on the levees and in the marshes by using cloth flags and preserved in 96% ethanol. All ticks were identified by using standard taxonomic keys (*12*). *A. triste* ticks were the only ticks collected from vegetation. For molecular analyses, individual specimens were removed from the ethanol solution, air-dried, and minced with a sterile scalpel blade. DNA was extracted by using a QIAamp DNA Mini-Kit (QIAGEN, Valencia, CA, USA) and eluted in a final volume of 100 μL. DNA extracts were evaluated by using a nested PCR designed to amplify a segment of the rickettsial outer membrane protein A gene (*ompA*) as described previously (*13*). In brief, 5 μL of each DNA extract was used as template with primers 190.70 and 190.701 in the primary reaction. Two microliters of each completed primary reaction was used as template with primers 190-FN1 and 190-RN1 in the nested reactions. Primers were used at a final concentration of 300 nmol/L in a 50-μL reaction mixture. All amplicons were sequenced and compared to those in GenBank by using the BLAST 2.0 program (http://blast.ncbi.nlm.nih.gov/blast.cgi). Separate laboratory rooms were used for extracting tick DNA, performing primary and nested PCRs, and sequencing reactions. Water blanks were used for each primary and nested assay, and all extracts that provided amplicons of the expected size were retested to confirm the result.

Amplicons were obtained from DNA extracts of 4 (5.8%) of 69 *A. triste* ticks collected from Reserva Natural Otamendi and 13 (8.4%) of 154 ticks collected from Estación Experimental INTA Delta del Paraná (Table). All 17 DNA samples produced amplicons of the expected sizes in primary reaction and nested reactions of the assay. Each 590-bp product (excluding primers) from the primary reaction was sequenced, and all sequences showed 100% identity with each other (GenBank accession no. FJ172358) and with the corresponding *ompA* sequence of *R. parkeri* (U43802).

**Table:** Prevalence of infection with *Rickettsia parkeri* in adult *Amblyomma triste* ticks collected in the lower Paraná River Delta, Buenos Aires Province, Argentina, 2007

Collection site	Month of collection	No. females (no. positive)	No. males (no. positive)	Total no. tested (no. positive)
Reserva Natural Otamendi	Jan	20 (0)	6 (0)	26 (0)
	Mar	5 (0)		5 (0)
	Jun	1 (0)		1 (0)
	Jul	2 (1)	3 (0)	5 (1)
	Nov	10 (0)	5 (0)	15 (0)
	Dec		17 (3)	17 (3)
Estación Experimental, Instituto Nacional de Tecnología Agropecuaria, Delta del Paraná	Aug	60 (5)	64 (3)	124 (8)
	Nov	15 (4)		15 (4)
	Dec	15 (1)		15 (1)
Total		128 (11)	95 (6)	223 (17)

## Conclusions

This study provides definitive evidence of *R. parkeri* in Argentina. Our findings have relevance for public health because the infected ticks were collected from the lower Paraná River Delta near the origin of several recently identified cases of eschar-associated rickettsiosis (*7*; Alfredo Seijo, pers. comm.). In Argentina, at least 15 species of hard ticks bite humans (*1*); however, the only *Amblyomma* tick reported to bite humans in the lower Paraná River Delta is *A. triste* (*2*). Our data suggest that *A. triste* ticks are vectors of *R. parkeri* in this region of Argentina. The prevalence of *R. parkeri*–infected *A. triste* ticks identified at these 2 locations is within the range of the infection prevalence of this agent reported in questing adult ticks collected in the state of São Paulo, Brazil (9.7%) and in Canalones County in southern Uruguay (2.6%) (*9**,**10*).

In South America, *R. parkeri* has been detected only in *A. triste* ticks (*9**,**10*), and in the United States, *R. parkeri* is found almost exclusively in *A. maculatum* ticks (*8**,**13*). *A. triste* and *A. maculatum* ticks are phylogenetically and morphologically similar, and *R. parkeri* appears to be strongly associated with these closely related tick species. Another human-biting Neotropical tick, *A. tigrinum*, is closely related to *A. triste* and *A. maculatum* ticks (*12*)*.* In this context, *A. tigrinum* ticks may also be involved in the transmission of *R. parkeri* in South America. The distribution of *A. triste* ticks extends from Argentina to Mexico, but this tick has been reported to bite humans only in a few regions of Argentina, Uruguay, and Venezuela (*1*). Because we are not aware of any records to indicate that immature stages of *A. triste* ticks will bite humans (*1**,**2**,**14*), our investigation focused on adult questing ticks for evidence of infection with *R. parkeri*. Preliminary studies indicate that peak adult *A. triste* abundance and activity in the lower Paraná River Delta occurs during August through November (S. Nava, unpub. data), similar to the seasonal distribution described for *A. triste* populations in southern Uruguay (*14*); most cases of eschar-associated disease in Argentina occur during this same interval (*7*; A. Seijo, pers. comm*.*)*.*

No immature *A. triste* ticks were collected by flagging during this investigation. However, larvae and nymphs were found at these study sites attached to the guinea pig (*Cavia aperea*) amd several species of sigmodontine rodents, including Azara’s grass mouse (*Akodon azarae*), the yellow pygmy rice rat (*Oligoryzomys flavescens*), the black-footed pygmy rice rat (*O. nigripes*), the red hocicudo (*Oxymycterus rufus*), and the Argentine swamp rat (*Scapteromys aquaticus*) (S. Nava, unpub. data*).* These findings suggest that one or more of these species may be involved in the natural transmission cycle of *R. parkeri* in this region.

Ecologic studies of *A. triste* ticks collected along the Paraná River in the states of São Paulo and Mato-Grosso do Sul in Brazil indicate that this tick is well-adapted to marsh habitats (*15*); the results of this investigation support this observation. The occurrence of an *R. parkeri* rickettsiosis-like disease in humans in the Paraná River Delta suggests that similar cases of human illness may occur in palustrine regions of other Central and South American countries where this tick is found. Additional studies are needed to better understand the natural history of *R. parkeri* in Argentina and in other countries of the Western Hemisphere.
